# 2-Deoxy-D-Glucose Treatment of Endothelial Cells Induces Autophagy by Reactive Oxygen Species-Mediated Activation of the AMP-Activated Protein Kinase

**DOI:** 10.1371/journal.pone.0017234

**Published:** 2011-02-28

**Authors:** Qilong Wang, Bin Liang, Najeeb A. Shirwany, Ming-Hui Zou

**Affiliations:** Section of Molecular Medicine, Department of Medicine, Department of Biochemistry and Molecular Biology, University of Oklahoma Health Sciences Center, Oklahoma City, Oklahoma, United States of America; University of Hong Kong, China

## Abstract

Autophagy is a cellular self-digestion process activated in response to stresses such as energy deprivation and oxidative stress. However, the mechanisms by which energy deprivation and oxidative stress trigger autophagy remain undefined. Here, we report that activation of AMP-activated protein kinase (AMPK) by mitochondria-derived reactive oxygen species (ROS) is required for autophagy in cultured endothelial cells. AMPK activity, ROS levels, and the markers of autophagy were monitored in confluent bovine aortic endothelial cells (BAEC) treated with the glycolysis blocker 2-deoxy-D-glucose (2-DG). Treatment of BAEC with 2-DG (5 mM) for 24 hours or with low concentrations of H_2_O_2_ (100 µM) induced autophagy, including increased conversion of microtubule-associated protein light chain 3 (LC3)-I to LC3-II, accumulation of GFP-tagged LC3 positive intracellular vacuoles, and increased fusion of autophagosomes with lysosomes. 2-DG-treatment also induced AMPK phosphorylation, which was blocked by either co-administration of two potent anti-oxidants (Tempol and N-Acetyl-L-cysteine) or overexpression of superoxide dismutase 1 or catalase in BAEC. Further, 2-DG-induced autophagy in BAEC was blocked by overexpressing catalase or siRNA-mediated knockdown of AMPK. Finally, pretreatment of BAEC with 2-DG increased endothelial cell viability after exposure to hypoxic stress. Thus, AMPK is required for ROS-triggered autophagy in endothelial cells, which increases endothelial cell survival in response to cell stress.

## Introduction

Autophagy is a tightly regulated catabolic process involving the degradation of cellular components using lysosomal machinery. This process plays an important role in cell growth, development, and homeostasis by maintaining a balance between the synthesis, degradation, and subsequent recycling of cellular products. Autophagy is a major mechanism by which a starving or stressed cell reallocates nutrients from ancillary processes to more essential ones [1]–[2]. For example, autophagy can be induced by hypoxia [3], energy deprivation [4], starvation [5] and ischemia [6]. Mechanistically, autophagy is initiated when the autophagosome, a double-membrane structure, is formed to surround certain targeted cytoplasmic proteins and organelles. This process and the double-membrane structures are associated with the conversion of the microtubule-associated protein light chain 3B-I (LC3-I) to LC3B-II. The protein/organelle containing autophagosome fuses with a lysosome to degrade its inner contents [1]. Lysosomes can be disrupted by chloroquine or bafilomycin A to block autophagosome degradation and provoke autophagosome accumulation, which is marked by an increase in LC3-II [7]. Increasing evidence suggests that autophagy plays an important role in the cardiovascular system under physiological and pathological conditions including ischemia-reperfusion injury in the heart and other organs [8], cardiomyopathy [9], myocardial injury, atherosclerosis [10], [11], and vascular pathology in Alzheimer's disease [12].

Reactive oxygen species (ROS) and reactive nitrogen species (RNS) are reported to be important in mediating autophagy [13], [14]. ROS have also been reported to stabilize autophagosomes during periods of nutrient deprivation, hypoxia, ischemia-reperfusion injury, and general cell stress [15]. For example, during cellular starvation or nutrient deprivation, increased generation of mitochondrial-derived hydrogen peroxide (H_2_O_2_) induces oxidation and consequent inhibition of Atg4, the cysteine proteases (autophagins) which play crucial roles in autophagy by proteolytic activation of Atg8 paralogs for targeting to autophagic vesicles by lipid conjugation, as well as in subsequent deconjugation reactions [16]. Despite of growing evidence that the redox regulation of the cysteine protease Atg4 by ROS correlates with the occurrence of autophagy, the mechanistic details of how ROS/RNS initiates autophagy remain to be elucidated.

AMPK is a serine/threonine kinase, which operates as a metabolic switch that is engaged in conditions when cellular ATP is becoming depleted. Upon activation, AMPK induces formation of the tuberous sclerosis complex to inhibit phosphorylation of the mammalian target of rapamycin (mTOR), which triggers autophagy through two downstream signaling partners, ribosomal protein S6 kinase and 4E-binding protein 1(4-eBP1) [17]. Some recent reports have implicated AMPK with regulation of autophagy. For example, aminoimidazole carboxamide ribonucleotide (AICAR) treatment and glucose deprivation of human mammary cancer derived cells (MCF-7s) inhibit autophagy [18]. Matsui and colleagues also reported that in cardiac myocytes, autophagy is induced by inhibition of mTOR, a phenomenon that protects against cell death [19].

Published studies from our laboratory and others have established an intricate balance between AMPK signaling and the redox state of vascular endothelial cells. ROS and RNS mediate AMPK activation induced by a wide range of stimuli, including hyperglycemia [20], hypoxia [21], treatment with metformin [22], nicotine [23], and therapy with statin drugs [24]. Conversely, AMPK activation inhibits the formation of ROS by NADPH oxidase and stimulates nitric oxide (NO) by endothelial NO synthase (eNOS) [25]. Further, AMPK has also been implicated in c-Jun N-terminal kinase (JNK) activation, nuclear factor (NF)- κB mediated transcription, E-selectin and vascular cell adhesion molecule-1 (VCAM-1) expression in endothelial cells [26]. However, whether or not AMPK is involved in ROS-triggered autophagy is unknown.

2-Deoxy-D-glucose (2-DG) is a relatively specific blocker for glycolysis because it cannot be further metabolized by hexokinase. Therefore, 2-DG triggers glucose deprivation without altering other nutrients or metabolic pathways [27]. In addition, 2-DG is reported to activate AMPK, increase ROS in cancer cells [28], and trigger autophagy [29]. Thus, 2-DG appears to be an ideal tool to dissect the interactions between autophagy, ROS, and AMPK. However, while 2-DG induces autophagy in cancer cells and mouse embryonic fibroblasts [30], [31], its ability to trigger autophagy in endothelial cells has not been demonstrated. In this study, we provide the first demonstration that AMPK is required for ROS-triggered autophagy in endothelial cells exposed to 2-DG.

## Materials and Methods

### Materials

Bovine aortic endothelial cells (BAEC) were obtained from Lonza Biologics, Inc. (Walkersville, MD). Human umbilical vein endothelial cells (HUVEC) were purchased from Cascade Biologics (Portland, OR). [γ-^32^P] was purchased from PerkinElmer Life Sciences (Waltham, MA). 2-DG, 4-hydroxy-Tempo, N-Acetyl-L-cysteine (NAc), ATP, ADP, AMP, allopurinol, and oxypurinol were purchased from Sigma-Aldrich (St. Louis, MO). Protein A/G-agarose, AMPKα siRNA, and UCP-2 siRNA were obtained from Santa Cruz Biotechnology, Inc. (Santa Cruz, CA). 5 (and-6)-chloromethyl-2′,7′-dichlorofluorescein diacetate, acetyl ester (CM-H_2_DCFDA), and Lipofectamine 2000 reagent were obtained from Invitrogen Corp. (Carlsbad, CA). Antibodies against phospho-acetyl-CoA carboxygenase (ACC; Ser79), phospho-AMPK (Thr172), phosphor-mTOR (Ser2448), AMPK, LC3B, and β-actin were purchased from Cell signaling Technology, Inc. (Beverly, MA). Antibody against UCP-2 was obtained from Santa Cruz Biotechnology, Inc.

### Cell Culture

BAEC and HUVEC were grown in endothelial cell basal medium (EBM) containing fetal bovine serum (5%, v/v), growth factors (Lonza) and 1% antibiotics (Invitrogen). BAEC and HUVEC were both cultured at 37°C in a 5% CO_2_ humidified atmosphere. Confluent BAEC and HUVEC were studied between passages 3 and 9.

### Adenoviral Infection

Proliferating BAEC at 60% confluence in a 6-well plate were inflected with adenovirus encoding super oxide dismutase 1 (SOD1), catalase, MnSOD, AMPK-dominant negative mutants (AMPK-DN), p47^phox^-domitant negative mutants (p47-DN), or p67^phox^ -dominant negative mutants (p67-DN). Adenovirus expression of green fluorescence protein (GFP) was used as negative control. After 48 hrs, the infection efficiency exceeded 80% as determined by the number of GFP-expressing cells.

### siRNA Transfection

Control non-targeted siRNA, AMPKα, or UCP-2 targeted siRNA (10 µM) were added to OPTI-MEM reduced serum media (GIBCO, Invitrogen) with Lipofectamine^TM^ 2000. Proliferating HUVEC at 60% confluence in a 6-well plate were incubated in 1.0 mL EBM supplemented with siRNA for 6 hrs. Then 1.0 mL EBM containing 2X fetal bovine serum and antibiotics was added, and the cells cultured for 24 hrs.

### Determination of ROS

BAEC were incubated in EBM without phenol red containing CM-H_2_DCFDA (5 µM) as a membrane-permeable probe to detect intracellular ROS, under various stimulation conditions. After 30 min incubation, fluorescence was detected using a Synergy HT microplate reader (BIO-TEK) with excitation set at 490 nm and emission detected at 520 nm.

### Measurement of AMP, ADP, and ATP

AMP, ADP, and ATP in BAEC were assessed by high performance liquid chromatography (HPLC) as described previously [32].

### AMPK Activity Assay

AMPK activity was determined using the SAMS peptide as an AMPK substrate, as previously described [22].

### Western Blotting and Quantification

Western blot was performed as described previously [22]. Band intensity (area × density) on Western blots was analyzed by densitometry (model GS-700, Imaging Densitometer; Bio-Rad, Hercules, CA). Background was subtracted from the quantification area.

### Light Microscopy and Immunofluorescence

Confluent BAEC on glass slides were transduced with LC3B-GFP encoding BacMam virus (Invitrogen) for 24 hrs. BAEC were then incubated for 0.5 hr with 1 µM LysoTracker Red (Invitrogen) and rinsed several times with PBS. The cells on the slides were fixed with 4% formaldehyde for 10 min and permeablized with 0.2% triton X-100 for 20 min. The slides were mounted and observed with OLYMPUS IX71 microscope (Olympus, Tokyo, Japan).

### Statistical Analysis

Data are presented as mean ± standard error of the mean (SEM). Differences were analyzed by using a one-way analysis of variance (ANOVA) followed by a Tukey post-hoc analysis. *p* values less than 0.05 were considered statistically significant.

## Results

### 2-DG induces autophagy in endothelial cells

**Figure 1:**
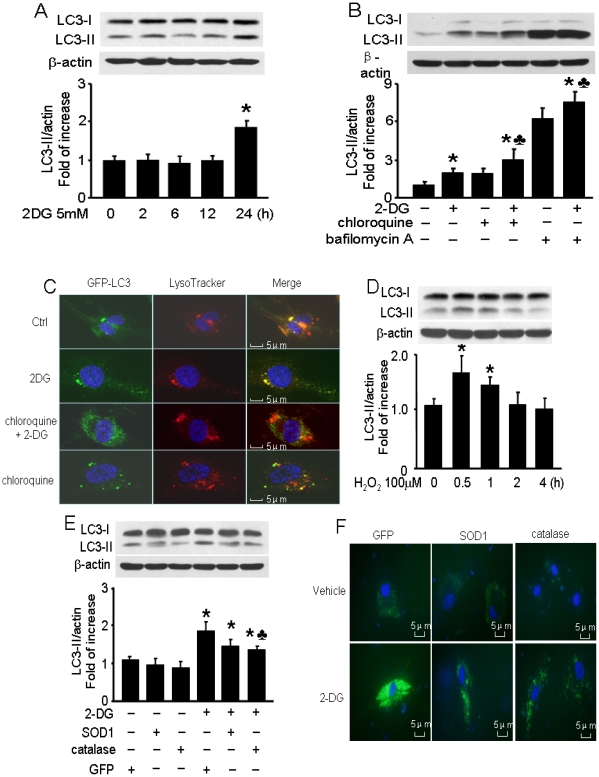
2-DG-induced autophagy is ROS-dependent. A: Confluent monolayers of BAEC were treated with 5 mM 2-DG for the indicated times. Cell lysates were analyzed by Western blot using antibody against LC3B. (n = 3; one-way ANOVA: *p<0.05 vs control). B: Confluent monolayers of BAEC were treated with 5 mM 2-DG for 24 hrs in the presence or absence of chloroquine (3 µM) or bafilomycin A (10 nM). Cell lysates were analyzed by Western blot for detection of LC3. (n = 3; two-way ANOVA: *p<0.05 2-DG vs vehicle, 2-DG + chloroquine vs. chloroquine, 2-DG + bafilomycin A vs. bafilomycin A. p<0.05, 2-DG vs. 2-DG + bafilomycin A or 2-DG + chloroquine). C: BAEC expressing GFP-LC3 were treated with chloroquine or 2-DG, and the accumulation of LC3 II (green), localization of LC3 II with lysosomes (red), and DAPI (blue) staining of nuclei in response to treatment were analyzed by fluorescence microscopy. Scale bars, 5 µm. D: Confluent monolayers of BAEC were treated with 100 µM H_2_O_2_ for the indicated times. Cell lysates analyzed by Western blot using antibody against LC3 (n = 3; one-way ANOVA: *p<0.05 vs control). E: BAEC were transduced adenovirus vectors encoding SOD1 or catalase for 48 hrs and then exposed to 5 mM 2-DG for 24 hrs (n = 3; two-way ANOVA, * p<0.05, GFP vs. 2-DG, SOD1 vs 2-DG + SOD1, catalase vs 2-DG + catalase, p<0.05, 2-DG vs. 2-DG + catalase overexpression). F: The accumulation of LC3 II (green), localization of LC3 II lysosomes (red), and DAPI (blue) staining of nuclei in response to SOD1 or catalase overexpression and 2-DG treatment were analyzed by fluorescence microscopy.

To establish whether 2-DG exposure triggered autophagy in endothelial cells, BAEC were exposed for 2 to 24 hrs to 5 mM 2-DG, a dose comparable to the physiologic concentration of D-Glucose, and the conversion of LC3-I to LC3-II was then determined. Exposure of BAEC to 2-DG for 24 hrs markedly increased the conversion of the cytoplasmic LC3-I to the autophagosomal membrane-bound LC3-II, indicating 2-DG might induce autophagy (Fig. 1A). However, rather than inducing autophagy, an increase in LC3-II could instead be due to 2-DG repressing autophagosome fusion with lysosomes and degradation of LC3-II. To determine the mechanism of 2-DG action, we examined the effect of disrupting lysosomal function using chloroquine and bafilomycin A. These compounds increase lysosomal pH and interfere with the function of lysosomal enzymes [7], thereby increasing autophagosome accumulation in the cell. BAECs treated with 2-DG and chloroquine or bafilomycin A had higher levels of LC3-II than cells treated with 2-DG alone (Fig. 1B), which indicates 2-DG acts by inducing autophagosome formation and does not disrupt their downstream maturation into autophagolysosomes.

To confirm the effect of 2-DG on autophagosome formation, immunofluorescence analysis of BAEC expressing GFP-LC3 and stained with Lysotracker (an organelle-selective, fluorescent probe that labels and tracks acidic organelles, such as lysosomes) was preformed. 2-DG increased formation of autophagosomes, indicated by increase in the number and distribution of GFP-tagged LC3 spots (Fig. 1C). Treatment of cell with both 2-DG and chloroquine further increased the number and distribution of LC3-GFP spots in these cells. Further, 2-DG increased fusion of GFP-LC3 tagged vacuoles with lysosomes, which is indicated by co-localization of GFP-LC3 with LysoTracker stained vesicles (Fig. 1C, merged image). This demonstrated that 2-DG treatment increased fusion of autophagosomes with lysosomes, a definitive event in the induction of cellular autophagy. Together, these data show that 2-DG induces conversion of LC3-I to LC3-II and triggers autophagy in endothelial cells.

### H_2_O_2_ mediates 2-DG-induced autophagy

Current literature indicates that H_2_O_2_ induces autophagy in HeLa cells and HEK293 cells [14], [33]. These findings are intriguing because nutrient starvation (such as 2-DG treatment) is known to generate ROS in a variety of cell types. We therefore tested whether H_2_O_2_ could also trigger autophagy in endothelial cells. Treatment of BAEC for 30 min with H_2_O_2_ (100 µM) increased the LC3-II-to-actin ratio 1.6-fold (Fig. 1D). We also reasoned that blocking the effects of ROS should mitigate the phenotype of 2-DG-induced autophagy. We tested this hypothesis by assessing the effect of 2-DG on the conversion of LC3I to LC3II in BAEC overexpressing either SOD1 or catalase from an adenovirus vector. SOD1 is an enzyme that buffers O_2_^−^ by converting it to H_2_O_2_ and catalase then scavenges H_2_O_2_ (Figure S1 in Text S1). Both SOD1 and catalase overexpression decreased 2-DG-enhanced LC3-II conversion. 2-DG treatment of cells expressing the GFP negative control had a 2.0-fold increase in LC3-II conversion, which was higher than the 1.5-fold increase observed in SOD1-overexpressing cells and 1.4-fold increase in catalase-overexpressing cells (Fig. 1E). To further elucidate the effect of antioxidant enzyme overexpression on 2-DG-induced autophagosome formation, immunofluorescence analysis of BAEC expressing GFP-LC3 was preformed. 2-DG treatment (lower three panels) increased formation of autophagosomes (green fluorescent spots) compared to non-2-DG treated cells (Fig. 1F). However, SOD1 and catalase overexpression attenuated the 2-DG-mediated formation of autophagosomes. Overall these results indicate that ROS are critically involved in 2-DG-induced autophagy and that H_2_O_2_ treatment of endothelial cells induces autophagy.

### 2-DG induced AMPK activation in a dose- and time-dependent manner

**Figure 2:**
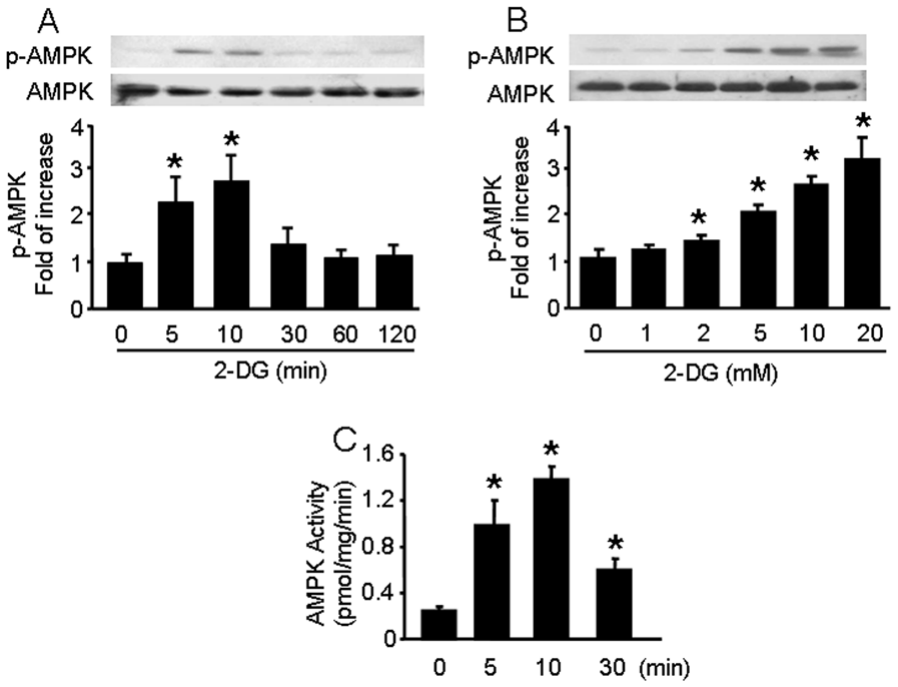
Time-course and dose-response for 2-DG-induced AMPK activation. A: Confluent BAEC monolayers were treated with 2-DG (5 mM) for the indicated times. Cell lysates were analyzed by Western blot using antibody against p-AMPK and AMPKα1/2 (n = 3; one-way ANOVA: *p<0.05 vs. control). B: Confluent BAEC monolayers were treated with the indicated concentration of 2-DG for 10 min (n = 3; one-way ANOVA: *p<0.05 vs. control). C: BAEC were incubated with 5 mM of 2-DG for the indicated times. AMPK was immunoprecipitated from cell lysis with an antibody against AMPKα bound to Protein A/G agarose overnight at 4°C. AMPK activity was determined by SAMS phosphorylation using a [^32^P]ATP assay (n = 3; one-way ANOVA, *p<0.05 vs. control).

To further establish if ROS is responsible for 2-DG-induced AMPK activation, we performed a time-course study to determine if ROS production proceeds AMPK activation. Confluent BAEC were stimulated with 5 mM 2-DG for 5 to 120 min, and AMPK activation was measured by monitoring phosphorylation of AMPKα-Thr172. As depicted in Fig. 2A, 2-DG treatment increased the phosphorylation of AMPKα-Thr172 within 5 min, and this reached a maximum of 2.8-fold greater than that in 2-DG untreated cells by 10 min after treatment without affecting the total AMPK levels. After 10 min, phosphorylated AMPKα-Thr172 levels decreased and returned to basal levels by 60 min after treatment. ACC is a well-established downstream target of AMPK and is activated by phosphorylation of Ser79. We measured ACC-Ser79 phosphorylation and observed a similar time course for ACC activation as that for AMPK (Figure S1 in Text S1). Further, 2-DG increased the phosphorylation of AMPK-Thr172 (Fig. 2B) and ACC-Ser 79 (Figure S1 in Text S1) in a dose-dependent manner. 2-DG also increased AMPK activity to 4-fold after 5 min compared to basal activity and reached a peak 5.5-fold higher than basal activity after 10 min (Fig. 2C). These data establish that 2-DG induces AMPK activation in both a time- and dose-dependent manner.

### AMPK is required for 2-DG-induced autophagy

**Figure 3:**
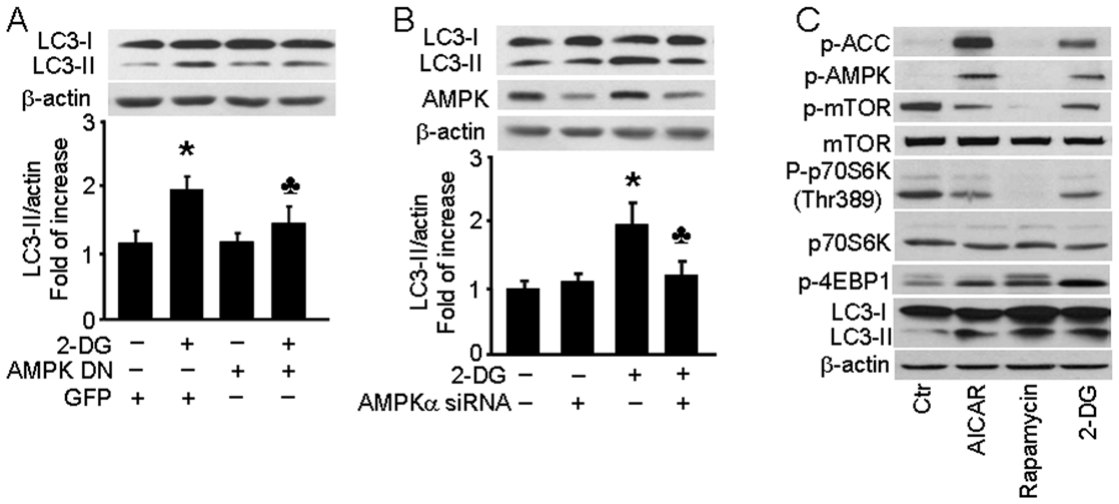
2-DG-induced autophagy is AMPK-dependent. A: BAEC were transduced with adenovirus vectors encoding dominant negative AMPK (AMPK-DN) for 48 hrs and then treated with 5 mM 2-DG for 24 hrs (*n* = 3; two-way ANOVA, * *p*<0.05, GFP *vs.* 2-DG, *p*<0.05, 2-DG *vs.* 2-DG + AMPK-DN). B: HUVEC were transfected with AMPK-targeted siRNA or control siRNA for 24 hrs then treated with 5 mM 2-DG for 24 hrs. Cell lysates were analyzed by Western blot using antibody against LC3 (*n* = 3; two-way ANOVA, * *p*<0.05, vehicle *vs.* 2-DG, *p*<0.05, Control siRNA *vs.* AMPK siRNA). C: BAEC were treated with 1 mM AICAR, 10 nM rapamycin, or 5 mM 2-DG for 24 hrs and then analyzed by Western blot to determine the level of phospho-AMPK-Thr172 (p-AMPK), phos-ACC-Ser79 (p-ACC), phos-mTOR-Ser2448 (p-mTOR), mTOR, phos-p70S6K-Thr389 (p-p70S6K), p70S6K, phos-4EBP1-Thr37/46 (p-4EBP1), and LC3.

In cancer cells, AMPK is reported to be a key regulator of autophagy via a mechanism that involves inactivation of mTOR [30] and cardiac myocytes [31]. We therefore explored whether AMPK was also involved in 2-DG-induced autophagy in endothelial cells. BAEC were transduced with an adenovirus vector encoding a dominant-negative AMPK (AMPK-DN) to block cellular AMPK functions. 2-DG treatment of cells expressing the GFP negative control for 24 hrs induced a 2-fold increase in LC3-II levels, but AMPK-DN overexpression blocked the 2-DG-induced increase in LC3-II levels (Fig. 3A). We confirmed these results by siRNA-mediated silencing of the AMPKα gene, which is the critical catalytic subunit of AMPK in HUVEC (Fig. 3B). As observed in BAEC, 2-DG treatment increased LC3-II levels 2-fold by 24 hrs post treatment in cells transfected with non-targeted control siRNA, but transfection with AMPKα1/2-targeted siRNA dramatically inhibited AMPK expression and reduced the 2-DG-induced increase in the LC3-II levels to 1.3-fold (Fig. 3B). Taken together, these data show that AMPK is required for 2-DG-induced autophagy in endothelial cells.

### AMPK induces autophagy *via* downstream mTOR signaling

The mTOR integrates the input from several upstream pathways by sensing the nutrient levels, bioenergetic status, and redox state of the cell [34]. We hypothesized that mTOR signaling was involved in the 2-DG induced AMPK-mediated induction of autophagy. We therefore examined phosphorylation status of AMPK and mTOR after AICAR, 2-DG, and rapamycin treatment. The 70-kDa ribosomal protein S6 kinase (p70S6K) and eukaryote initiation factor 4E binding protein 1 (4E-BP1) are the two well-characterized targets of mTORC. When we probed BAEC by Western blot analysis, we observed that both AICAR and 2-DG led to the phosphorylation of AMPK as well as its upstream activator ACC (Fig. 2C). As predicted, both AICAR and 2-DG also attenuated the phosphorylation of mTOR and its two targets, p706K and 4E-BP1. Similarly, the canonical mTOR inhibitor rapamycin increased of LC3-II levels without affecting AMPK phosphorylation (Fig. 2C and Figure S2 in Text S1). Thus, we concluded that 2-DG induced autophagy *via* activation of AMPK, which functions through the downstream inhibition of the mTOR signaling pathway.

### ROS is involved in 2-DG-induced AMPK activation, which can be attenuated by anti-oxidant treatment

**Figure 4:**
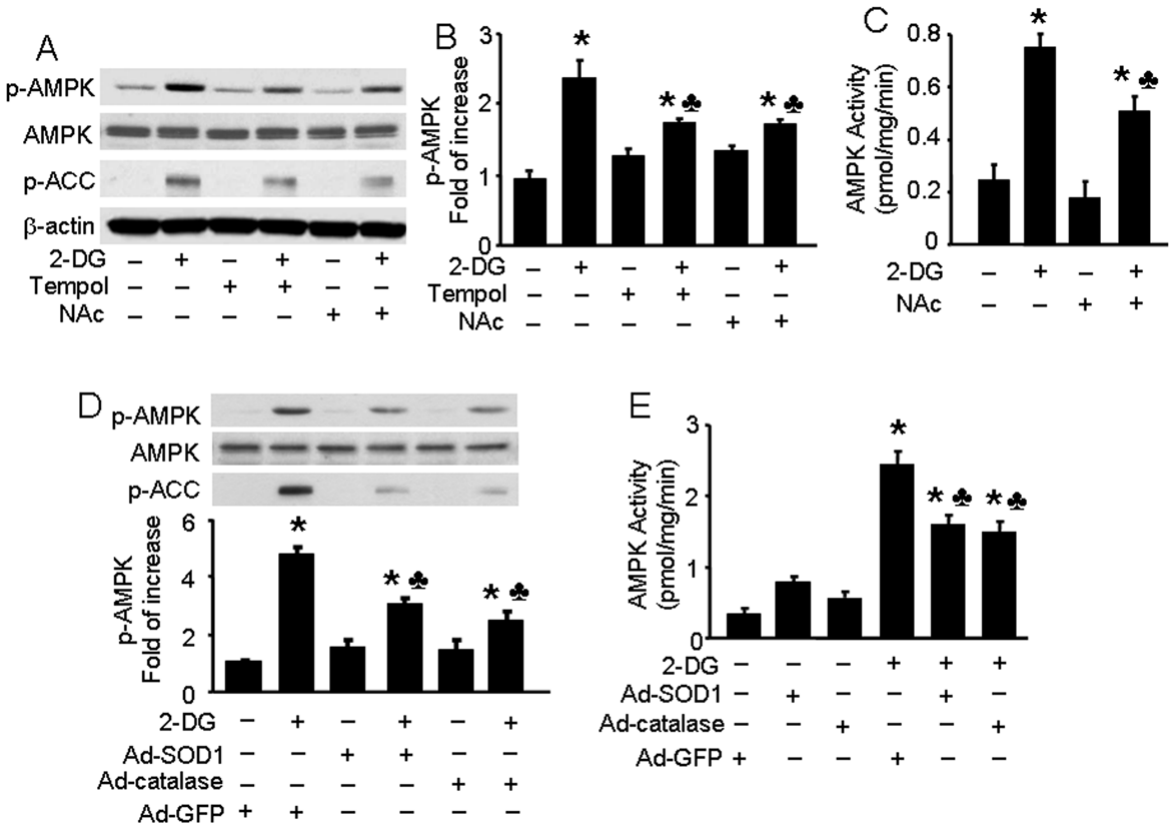
Inhibitory effects of antioxidants on 2-DG-induced AMPK activation in BAEC. A, B: Confluent BAEC monolayers were pretreated with 10 µM 4-hydroxy-Tempol (Tempol) or 2 mM N-Acetyl-Cysteine (NAc) for 30 min and then treated with 5 mM 2-DG for 10 min. Cell lysates were subjected to Western blot analysis using antibodies against p-AMPK, p-ACC, AMPKα1/2, and β-actin (*n* = 3; two-way ANOVA,* *p*<0.05, 2-DG *vs.* control, Tempol + 2-DG *vs.* Tempol, NAc + 2-DG *vs.* Nac, *p*<0.05 2-DG *vs* 2-DG + Tem or NAc). C: BAEC were pretreated with NAc and then stimulated with 2-DG. AMPK activity was determined by SAMS phosphorylation using a [^32^P]ATP assay (*n* = 3; two-way ANOVA, * *p*<0.05, vehicle *vs* 2-DG, Nac *vs* 2-DG + NAc, *p*<0.05 2-DG *vs.* 2-DG + NAc). D, E: Confluent BAEC monolayers were transduced with adenovirus vectors encoding SOD1 or catalase for 48 hrs and then treated with 5 mM 2-DG for 10 min. Phosphorylation of AMPK and AMPK activity were detected, as described in the Matierals and Methods (*n* = 3; two-way ANOVA, * *p*<0.05, GFP *vs.* 2-DG, SOD1 *vs* 2-DG + SOD1, catalase *vs* 2-DG + catalase, *p*<0.05, 2-DG *vs.* 2-DG + SOD1 or catalase overexpression).

Recent studies using cancer cell lines [28] and *C. Elegans* [35] showed that glucose deprivation activated AMPK by production of ROS. However, it is not clear whether ROS also regulate 2-DG-induced AMPK activation in endothelial cells. To evaluate this, we first examined the effects of antioxidants on 2-DG-induced AMPK activation in BAEC. Pre-treatment of BAEC with Tempol (an O_2_^−^ scavenger; 10 µM) or NAc (a thiol antioxidant; 2 mM) significantly reduced 2-DG-enhanced phosphorylation of AMPK. In addition, NAc inhibited ACC phosphorylation induced by 2-DG (Fig. 4A and B, Figure S2 in Text S1). We further assayed AMPK activity in BAEC by quantifying phosphorylation of the SAMS peptide, an AMPK substrate, using a [^32^P]-ATP assay. After treatment with 2-DG (5 mM), AMPK activity was 3-fold greater than baseline within 10 min, and NAc treatment caused AMPK activity to drop from 3-fold to 2-fold over baseline levels (Fig. 4C). Finally, we overexpressed antioxident proteins SOD1 or catalase to further examine the involvement of ROS (Figure S3 in Text S1). Overexpression of either SOD1 or catalase, but not GFP, attenuated 2-DG-enhanced phosphorylation of AMPK and ACC (Fig. 4D). In the SAMS peptide phosphorylation assay, 2-DG treatment increased AMPK activity 7-fold, and this was enhanced 2-fold by overexpression of SOD1 and 2.5-fold by overexpression of catalase (Fig. 4E). Overall these results indicate that ROS are involved in 2-DG-induced AMPK activation in endothelial cells, a phenomenon that is reversible if ROS levels are suppressed with anti-oxidants.

### 2-DG increases intracellular H_2_O_2_ levels in endothelial cells

**Figure 5:**
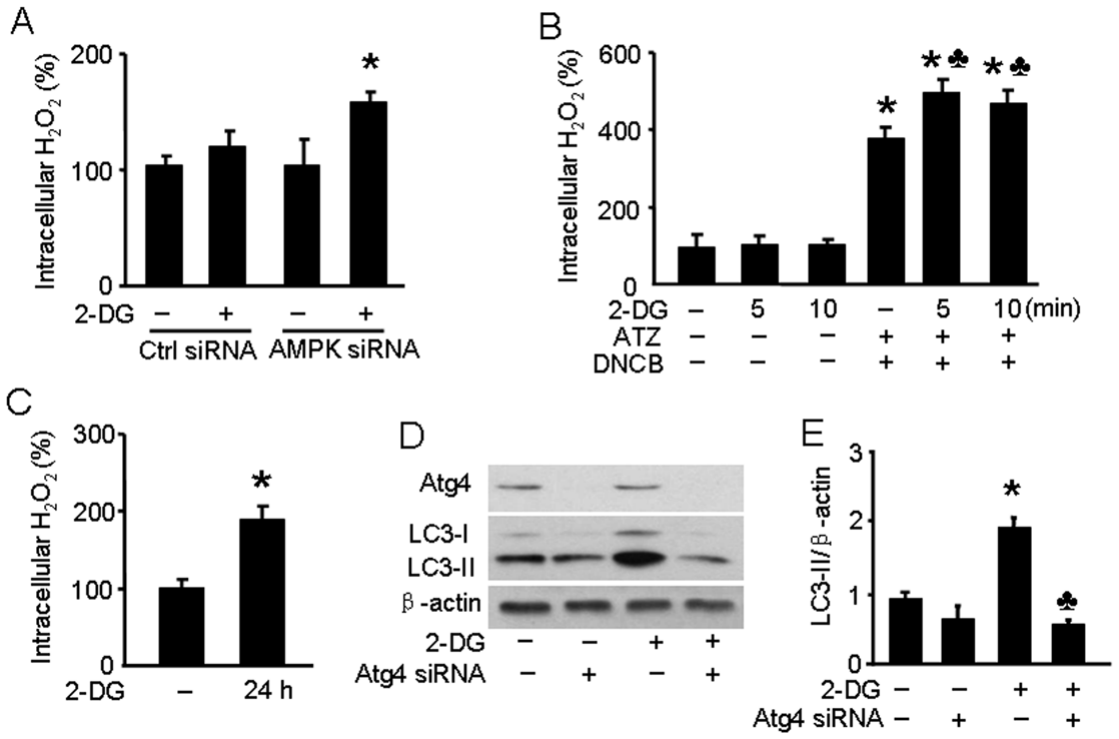
2-DG increases intracellular synthesis of H_2_O_2_ to induce autophagy. A: HUVEC were transfected with AMPK-targeted siRNA for 24 hrs. Then the cells were incubated in EBM media without phenol red and treated with 5 mM of 2-DG for 10 min. CM-H_2_DCFDA (10 µM; Invitrogen) was added for 30 min before quantification of fluorescence (excitation at 485 nm and emission at 545 nm). (*n* = 3; two-way ANOVA,* *p*<0.05, 2-DG *vs.* vehicle). B: BAEC were pre-treated with 3-amino-1,2,3-triazine (ATZ, 100 µM) and 1-chloro-2,4-dinitrobenzene (DNCB, 100 µM) for 30 min and then treated with 2-DG for 5 or 10 min. Intracellular H_2_O_2_ levels were detected by fluorescence using CM-H_2_DCFDA. (*n* = 3; two-way ANOVA,* *p*<0.05, ATZ + DNCB *vs.* vehicle, *p*<0.05, 2-DG + ATZ + DNCB *vs.* ATZ + DNCB). C: BAEC were treated with 2-DG for 24 hrs. Intracellular H_2_O_2_ levels were detected by fluorescence using CM-H_2_DCFDA. (*n* = 3; t-test,* *p*<0.05, 2-DG *vs.* vehicle). D,E: HUVEC were transfected with Atg4-targeted siRNA or control siRNA for 24 hrs and then treated with 5 mM 2-DG for 24 hrs. Cell lysates were analyzed by Western blot using antibody against LC3 (*n* = 3; two-way ANOVA, * *p*<0.05, vehicle *vs.* 2-DG, *p*<0.05, Control siRNA *vs.* Atg4 siRNA).

We next determined the effect of 2-DG on ROS production in endothelial cells using CM-H_2_DCFDA as an intracellular H_2_O_2_ probe. It is known that AMPK activation by AICAR reduces the generation of ROS [20]. To circumvent the confounding potential of ROS attenuation by AMPK activation, we detected intracellular H_2_O_2_ in 2-DG-stimulated HUVEC transfected with AMPK-targeted siRNA. As expected, the intracellular H_2_O_2_ levels in cells in which AMPKα1/2 was knocked down were significantly higher than in cells transfected with the non-targeted control siRNA following a 10–min, 5-mM 2-DG treatment (Fig. 5A). Pre-treatment with a catalase inhibitor 3-amino-1,2,4-triazine (ATZ) and thioredoxin reductase inhibitor 1-chlo-2,4-dinitrobenzene (DNCB) led to a significantly increased intracellular H_2_O_2_ level in 2-DG-treated BAEC (Fig. 5B and C).

### Suppression of Atg4 attenuates 2-DG induced autophagy

Sherz-Shouval et al. [16] recently reported that ROS are essential for autophagy and specifically regulate the activity of Atg4. Although these studies were done in Chinese Hamster Ovary and HeLa cell lines, it is likely that these results can be extrapolated to endothelial cells as well. Based on these findings, we examined Atg4 expression in HUVEC after 2-DG treatment or after transfection of Atg4-targeted siRNA. As shown in Fig. 5D, silencing of Atg4 efficiently blocked Atg4 expression in HUVEC. Further, 2-DG treatment of Atg4-silenced cells led to an attenuated increase in LC3-II levels. These findings are consistent with the observation that H_2_O_2_ inhibits Atg4, which in turn promotes lipidation of Atg8 leading to increased autophagy.

### ROS-dependent AMPK activation is independent of changes in the AMP-to-ATP ratio

**Figure 6:**
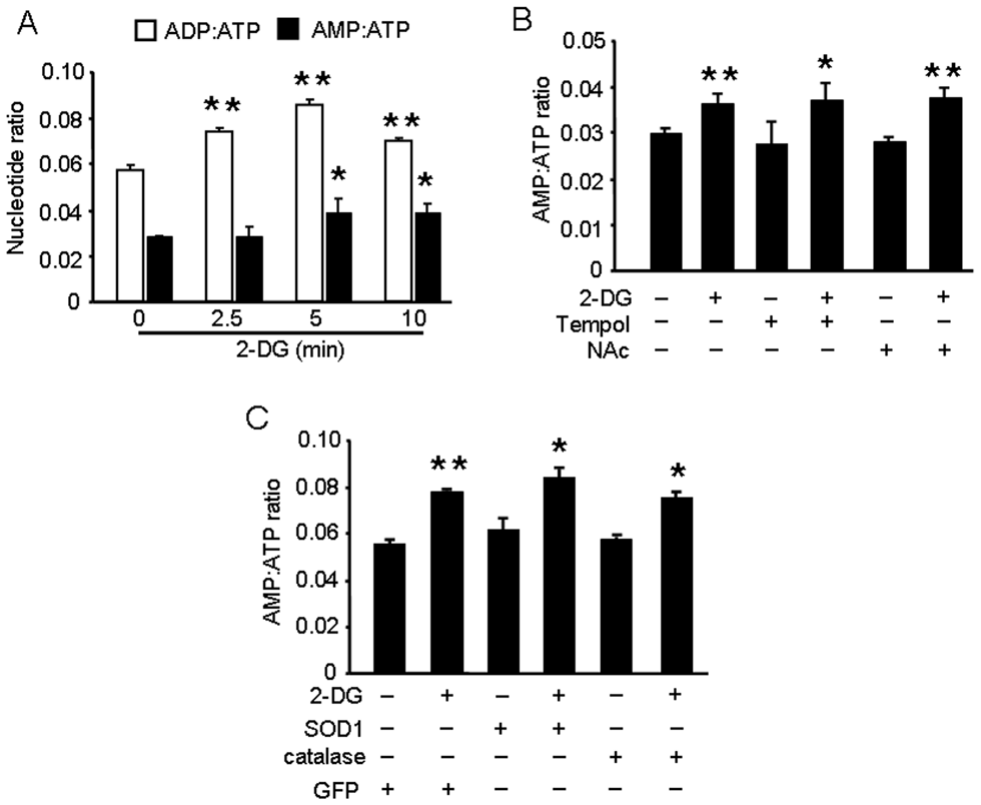
Inhibition of AMPK by antioxidants is independent of the AMP:ATP ratio. A: Confluent BAEC monolayers were treated with 5 mM of 2-DG for the indicated times. Cells were lysed by perchloric acid and centrifuged as described in the Materials and Methods. The ultrafiltrate was injected into a Jasco HPLC, and ATP, ADP, and AMP were monitored at 260 nm. (n = 4; one-way ANOVA, * *p*<0.05, ** *p*<0.01, Control vs. 2-DG treatment). B: BAEC monolayers were pre-treated with 10 µM Tempol or 2 mM NAC and then treated with 5 mM of 2-DG for 5 min. The AMP:ATP ratio in cell lysates was measured by HPLC (*n* = 5; two-way ANOVA,* *p*<0.05, ** *p*<0.01, 2-DG *vs.* vehicle, Tempol *vs.* 2-DG + Tempol, NAc *vs.* 2-DG + NAc). C: BAEC overexpressing SOD1 or catalase were treated with 5 mM 2-DG for 5 min. The AMP:ATP ratio in cell lysates was measured by HPLC. (*n* = 3; * *p*<0.05, ** p<0.01, two-way ANOVA, GFP *vs.* 2-DG, SOD1 *vs.* 2-DG + SOD1, catalase *vs.* 2-DG + catalase).

Scott et al. found that there are regulatory AMP- and ATP-binding sites in the subunits of AMPK [36]. As a consequence, high concentrations of ATP antagonize AMPK activation that results from AMP binding to the heterotrimer. 2-DG appears to induce AMPK activation primarily through the decrease in intracellular ATP levels that results from blocking glycolysis. We speculated that ROS-mediated AMPK activation via glucose deprivation might not depend on a change in the AMP-to-ATP ratio. We therefore quantified the ADP:ATP and AMP:ATP ratios in BAEC exposed to 2-DG at different time points after treatment. Both AMP:ATP and ADP:ATP ratios significantly increased by 5 min after 2-DG addition (Fig. 6A). Neither pretreatment of BAEC with Tempol or NAc (Fig. 6B) nor overexpression of SOD1 or catalase inhibited the observed increase in these ratios (Fig. 6C). This is the first study to demonstrate that 2-DG-induced AMPK activation is mediated by ROS and is likely independent of the AMP:ATP nucleotide ratio.

### Mitochondria are the sources of ROS in 2-DG-treated endothelial cells

**Figure 7:**
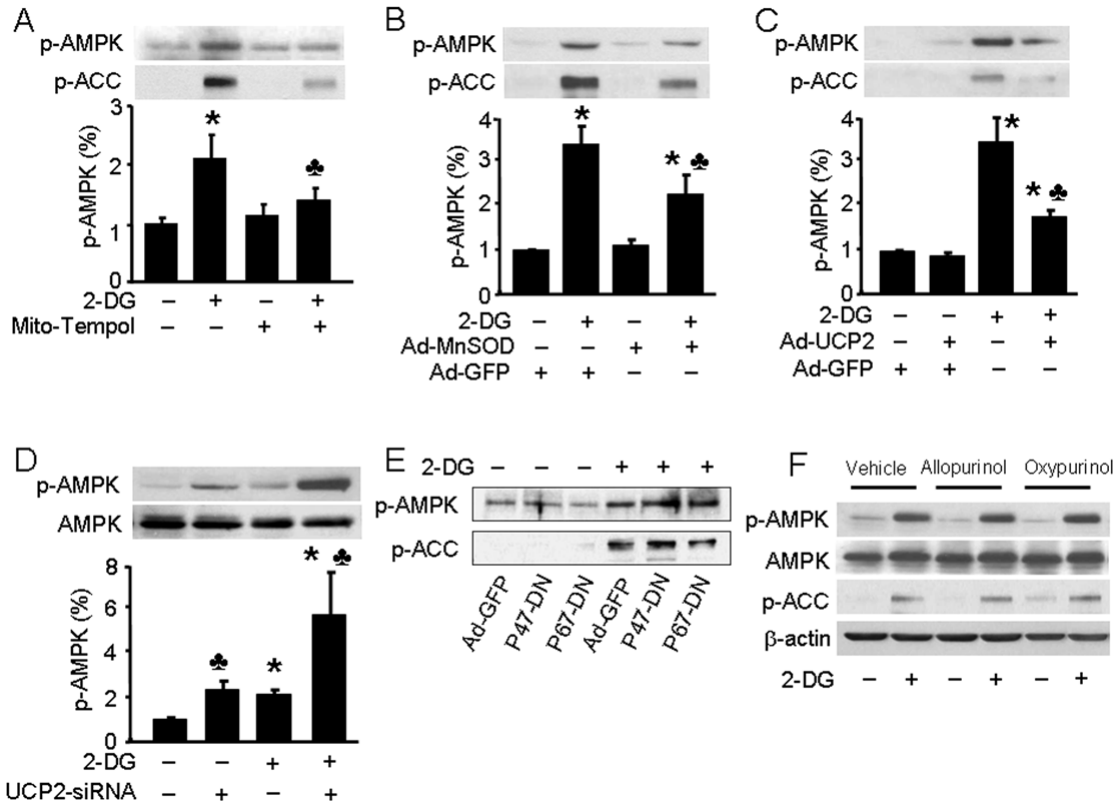
Activation of AMPK by 2-DG is mediated by mitochondrial ROS independent of NAD(P)H oxidase and xanthine oxidase. A: Confluent BAEC monolayers were pre-treated with 10 µM mito-Tempol for 30 min and then treated with 5 mM of 2-DG for 10 min. Cell lysates were analyzed by Western blot using antibody against p-AMPK (*n* = 3; two-way ANOVA, *p<0.05, vehicle *vs* 2-DG, *p*<0.05, 2-DG *vs.* 2-DG + mito-Tem). B, C, E: Confluent BAEC monolayers were transduced with adenovirus vectors encoding MnSOD (B), UCP2 (C), or p47^phox^ and p67^phox^ dominant negative mutants (E) for 48 hrs and then treated with 5 mM 2-DG for 10 min. (*n* = 3; two-way ANOVA, * *p*<0.05, GFP *vs* 2-DG + GFP, MnSOD *vs* 2-DG + Mn SOD, UCP2 *vs* 2-DG + UCP2, *p*<0.05 GFP *vs.* MnSOD or UCP2). D: HUVEC were transfected with control siRNA or UCP-2-targeted siRNA for 24 hrs. Then the cells were treated with 5 mM of 2-DG for 10 min. (*n* = 3; two-way ANOVA, *p<0.05, 2-DG *vs* vehicle, *p*<0.05 control siRNA *vs.* UCP2 siRNA). F: Confluent BAEC monolayers were pre-treated with allopurinol (100 µM) or oxypurinol (30 µM) for 30 min and then treated with 5 mM of 2-DG for 10 min.

Mitochondria, NAD(P)H oxidase, and xanthine oxidase [37] can generate ROS in both physiological and pathological conditions. We sought to identify the major sources of ROS synthesis in 2-DG-treated endothelial cells. Mito-Tempol is a synthetic Tempol derivate that preferentially scavenges O_2_^−^ from mitochondria. Pre-treatment of cells with 10 µM mito-Tempol for 30 min dramatically decreased 2-DG-induced phosphorylation of AMPK (Fig. 7A). Further, overexpression of MnSOD, a SOD isoform located in the mitochondrial matrix (Figure S3 in Text S1), attenuated the 2-DG-induced phosphorylation of AMPK and ACC (Fig. 7B).

Mitochondrial uncoupling protein-2 (UCP-2) is a mitochondrial anion carrier protein that mediates mitochondrial proton leakage and uncouples ATP synthesis from oxidative phosphorylation. UCP-2 overexpression (Figure S3 in Text S1) dramatically decreased the 2-DG phosphorylation of AMPK and ACC (Fig. 6C). Consistently, transfection of UCP-2-targeted siRNA, but not control siRNA, enhanced both basal phospho-AMPK levels and 2-DG-induced AMPK phosphorylation in HUVEC (Fig. 7D and Figure S3 in Text S1). To further identify the sources of ROS in these cells, we used infected cells with adenovirus vectors carrying dominant negative subunits of NADPH oxidase to induce NAD(P)H oxidase dysfunction. p47^phox^ and p67^phox^ are subunits of the NADPH oxidase that activate NAD(P)H oxidase activity by binding with gp91^phox^ on the mitochondrial membrane [38], but expression of dominant negative p47^phox^ (p47-DN) and p67^phox^ (p67-DN) lead to NAD(P)H oxidase dysfunction. p47-DN or p67-DN overexpression did not affect 2-DG-induced AMPK and ACC phosphorylation (Fig. 7E and Figure S3 in Text S1). Moreover, incubation of BAEC with the xanthine oxidase inhibitors, allopurinol and oxypurinol, did not block 2-DG-induced AMPK and ACC phosphorylation (Fig. 7F and G). These findings strongly suggest that mitochondria are the main source of ROS induced by 2-DG in BAEC.

### AMPK-regulated autophagy contributes to endothelial cell survival under hypoxic conditions

**Figure 8:**
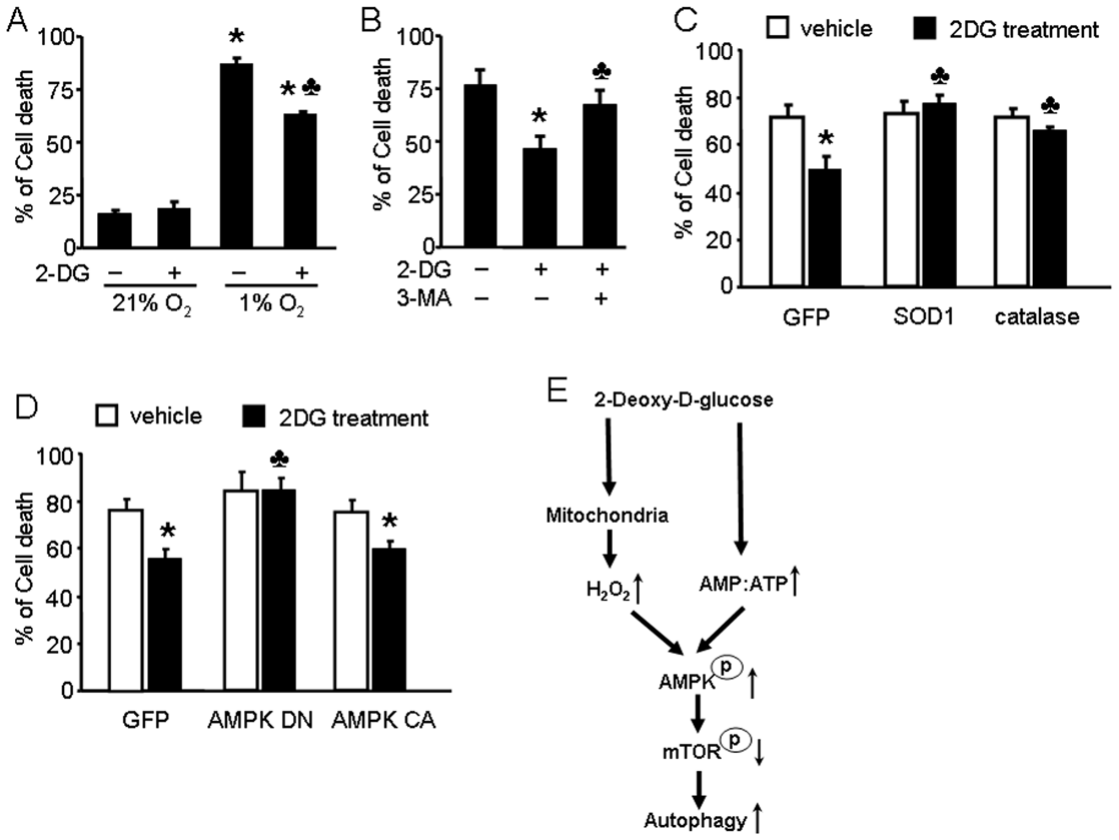
Inhibitory effect of 2-DG-induced autophagy on cell death during hypoxia is dependent on ROS and AMPK. A: BAEC cells were incubated in the absence 5 mM 2-DG for 24 hrs under normoxia (21% oxygen) or hypoxia (1% oxygen) conditions for 12 hrs. LDH in media was measured to assess cytotoxicity. (*n* = 4; two-way ANOVA, * *p*<0.05, normoxia *vs* hypoxia, *p*<0.05 vehicle *vs.* 2-DG treatment). B: BAEC were pretreated with 3-methyladenine (3-MA) for 0.5 hr and then treated with 5 mM 2-DG for 24 hrs. Then, BAEC were incubated in hypoxic conditions for 12 hrs. (*n* = 4; * *p*<0.05, two-way ANOVA, vehicle *vs.* 2-DG treatment, *p*<0.05, 2-DG *vs.* 2-DG + 3-methyladenine). C: BAEC were transduced with adenovirus vectors encoding SOD1 or catalase for 48 hrs and then treated with 5 mM 2-DG for 24 hrs. Then, BAEC were incubated in hypoxic conditions for 12 hrs. (*n* = 4; two-way ANOVA, *p<0.05, vehicle *vs.* 2-DG treatment, *p*<0.05, GFP + 2-DG *vs.* SOD1 or catalase overexpression + 2-DG). D: BAEC were transduced with adenovirus vectors encoding AMPK-DN or AMPK-CA for 48 hrs and then treated with 5 mM 2-DG for 24 hrs. Then, BAEC were incubated in hypoxic conditions for 12 hrs. (*n* = 4; two-way ANOVA, *p<0.05, vehicle *vs.* 2-DG treatment, *p*<0.05, GFP *vs.* AMPK-DN). E: The model for 2-DG-induced autophagy in endothelial cells. 2-DG increased H_2_O_2_ production by mitochondria and the AMP:ATP ratio in endothelial cells. H_2_O_2_ subsequently increased AMPK activation, independent of a change in the AMP:ATP ratio. ROS-dependent activation of AMPK by 2-DG is required for autophagy.

We next sought to demonstrate the physiological relevance of our findings. For this purpose, we exposed BAEC to hypoxic conditions and treated them with 2-DG to determine the role that autophagy plays under hypoxic conditions. As showed in Figure 8A, we observed increased cell death in BAEC after 12 hrs of hypoxia. Interestingly, 2-DG pre-treatment prevented the hypoxia-induced cell death. We quantified the release of lactate dehydrogenase (LDH) from cells as a marker of plasma membrane damage, reflecting the degree of cell death. LDH release, and therefore cell death, in BAEC pre-treated with 2-DG (5 mM) was significantly lower than that in untreated cells (Fig. 8A). In these experiments, we were also able to demonstrate that whereas 2-DG protected cells from hypoxia-induced death, pre-treatment with 3-methyladenine (3-MA), an autophagy inhibitor, partially blocked the 2-DG-induced protection (Fig. 8B). In contrast, SOD1 or catalase overexpression blocked the 2-DG-induced protection and cell survival under hypoxic conditions (Fig. 8C). Finally, AMPK-DN overexpression prevented 2-DG-induced cell survival (Fig. 8D). Together, these results show that 2-DG induces autophagy through AMPK activation in a ROS-dependent manner, thereby protecting cells from hypoxia-induced cell death.

## Discussion

The present study has, for the first time, demonstrated that AMPK activation by mitochondrial ROS is required for the induction of autophagy in endothelial cells. Mechanistically, we found that ROS, via AMPK activation, increase autophagy by inhibition of mTOR and Atg4 activity and ROS-triggered autophagy increases endothelial cell survival under hypoxic conditions. Thus, our results reveal a novel signaling pathway in which mitochondrial ROS activate AMPK that in turn increases autophagy. Induction of autophagy then serves to increase endothelial cell survival (Fig. 8E).

Glucose deprivation induces a depletion of intracellular ATP [39], which consequently elevates the AMP:ATP ratio and activates AMPK [29]. Recent study has demonstrated that glucose deprivation increases O_2_^−^ and H_2_O_2_ generation in human colon and breast cancer cells [40]. Our results demonstrate that 2-DG induces autophagy in endothelial cells, a phenomenon not previously reported in this cell type. Specifically, we have shown that whether AMPK is activated via nutritional stress (as in exposure to 2-DG) or by induction of ROS synthesis, endothelial cells activate an autophagy signaling pathway rather than a cell death pathway. This result is consistent with other published data. For example, Matsuda et al. reported that 2-DG induces autophagy in A/J mouse peritoneal macrophages [41]. This process is also likely to be involved in the ability of 2-DG to prevent brain injury induced by ischemia-reperfusion [42]. 2-DG is also reported to decrease phosphorylation of mTOR and its downstream targets, p70S6k and 4E-BP1, in human breast cancer cells, leading to a reduced carcinogenic response and attenuated tumorigenicity [43]. AMPK activation also represses mTOR activity in cells with reduced energy stores or after AICAR treatment [44]. Consistent with these findings, our results demonstrate that 2-DG induces autophagy through an AMPK-regulated pathway, and siRNA-mediated AMPK knockdown prevents conversion of LC3-I to LC3-II. Most compelling of all, in our validation of data showing the involvement of H_2_O_2_ in 2-DG-enhanced phosphorylation of AMPK, we were also able to demonstrate that ROS are involved in the coordinated role of 2-DG and AMPK on autophagy. Overall, these data indicate a novel autophagy signaling pathway important for endothelial cell survival.

Our results have further demonstrated that ROS might be a general mechanism for AMPK activation under certain physiological conditions, including glucose deprivation. Consistent with these findings, Cai et al. demonstrated that glucose deprivation induces ROS production to activate AMPK in pancreatic β cells [45]. Several groups, including our own, have previously shown that diverse stimuli can induce ROS generation, and in general, this results in AMPK activation. Exposure to different oxygen concentrations, ranging from frank hypoxia [46] to hyperemia [47], triggers AMPK activation *via* mitochondrial ROS production. In addition, many exogenous stimuli, such as free fatty acids, including palmitic acid and arachidonic acid [48], pharmacological stimuli, such as metformin [22], etoposide, and resveratrol have all been observed to increase AMPK activation through a mitochondrial ROS mechanism. All of these compounds are potentially important drug treatments. Metformin is a common treatment for diabetes and metabolic syndrome. Etoposide is a cancer therapeutic agent that inhibits topoisomerase II activity, and resveratrol is a botanical phytoallexin that has shown beneficial effects on hyperglycemia in animal models of diabetes [49]. Phosphorylation of AMPK via ROS is also seen to occur in skeletal muscle during both stretch [50] and contraction [51]. These data support our concept that AMPK is an important redox sensor that effectively operates in a variety of physiological and pathological conditions.

Another important finding in this study is that 2-DG-induced ROS-activated AMPK activation prevents mitochondrial ROS generation using a feedback system. Our data suggest an intricate balance between AMPK signaling and the redox state of vascular endothelial cells. AMPK activation maintains redox homeostasis by inhibiting intracellular ROS production from mitochondrial, NAD(P)H oxidase or by increasing anti-oxidant gene expression. Our group previously reported that AMPK activation up-regulates UCP-2 expression to prevent oxidant stress [52]. Additional indirect evidence of these redox regulating properties has been described. For example, AICAR induces AMPK activation and attenuates the ROS production seen in hyperglycemia [20]. Rosiglitazone, a thiozolidinedione drug, also induces AMPK activation and prevents hyperglycemia-induced ROS production [53]. AMPK activation can also inhibit ROS production from other sources, such as NAD(P)H oxidase and adiponectin exposure [54]. Moreover, AMPK has also been implicated in the regulation of anti-oxidant enzyme expression in endothelial cells by directly regulating the forkhead box O (FoxO) 1 and 3 transcription factors [55] to induce expression of thioredoxin (Trx), which is an important ROS scavenger. Indeed, investigators have also identified AMPK-FoxO-Trx as being involved in the cellular antioxidant defense mounted against disparate ROS/cell stress triggers, such as shear stress [56] and free fatty acid stimulation [48]. AMPK activation was also reported to increase the expression of MnSOD [57], and AMPKα1 deletion decreases the expression of SOD, catalase, γ-glutamylcystine synthase, and Trx [58]. Therefore, AMPK activation appears to be an important target for maintaining cellular redox homeostasis.

Endothelial dysfunction is one of the earliest events that occurs in a number of cardiovascular diseases, including atherosclerosis, hypertension, and diabetes. Endothelia dysfunction is characterized by accelerated endothelial cell death [59]. AMPK expression levels and activation are reduced in the early stages of these cardiovascular diseases [26], [60]. Thus, the defects in the ROS-AMPK-autophagy pathway described in these studies may contribute to the initiation and progression of endothelial dysfunction. Therefore, greater insight into the complex autophagic and apoptotic regulatory controls in the endothelium and vascular smooth muscle system is likely to lead to improved clinical treatments for related diseases.

## Supporting Information

Text S1 Supporting Figures S1, S2, and S3
(DOC)
