# Supplementary material for: 2-Deoxy-D-Glucose Treatment of Endothelial Cells Induces Autophagy by Reactive Oxygen Species-Mediated Activation of the AMP-Activated Protein Kinase
Source: PLoS One. 2011 Feb 28;6(2):e17234. doi: 10.1371/journal.pone.0017234 (PMC3046135; doi:10.1371/journal.pone.0017234)
Supplement: Text S1 — Supporting Figures S1, S2, and S3 (DOC) [file pone.0017234.s001.doc]

**Supplemental Data**

**2-Deoxy-D-glucose Treatment of Endothelial Cells Induces Autophagy by Reactive Oxygen Species-mediated Activation of the AMP-activated protein kinase**

Qilong Wang, Bin Liang, Najeeb A Shirwany and Ming-Hui Zou*


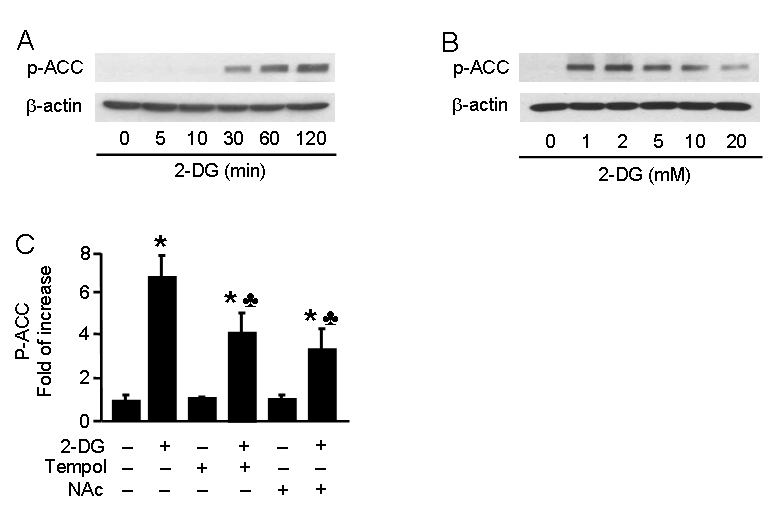


**Supplement Figure S1.**

**2-DG dose- and time-dependent induces phosphorylation of ACC.**

A: Confluent BAEC monolayers were treated with 2-DG (5 mM) for the indicated times. Cell lysates were analyzed by Western blot analysis using antibody against phospho-ACC. B: Confluent BAEC monolayers were treated with 1 mM to 20 mM 2-DG for 10 min. Cell lysates were analyzed by Western blot analysis using antibody against phospho-ACC-Ser^79^. C: Confluent BAEC monolayers were pretreated with 10 μM 4-hydroxy-Tempol (Tempol) or 2 mM NAc for 30 min and then treated with 5 mM 2-DG for 10 min. Cell lysates were analyzed by Western blot using antibody against p-ACC (*n* = 3; two-way ANOVA,* *p* < 0.05, 2-DG *vs.* control, Tempol + 2-DG *vs.* Tempol, NAc + 2-DG *vs.* Nac, ♣ *p* < 0.05 2-DG *vs* 2-DG + Tem or NAc).


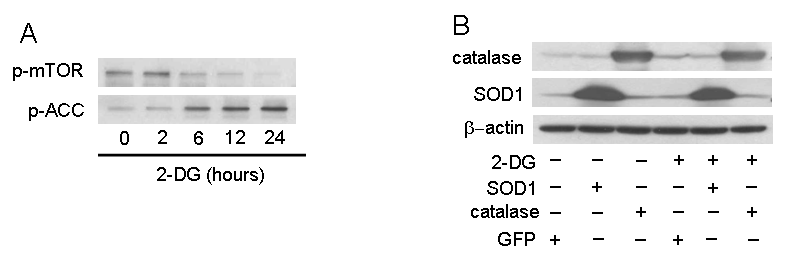


**Supplement Figure S2.**

**2-DG decreases phosphorylation of mTOR.** A: Confluents BAEC monolayers were treated with 5 mM 2-DG for the indicated times. Cell lysates were analyzed by Western blot analysis using antibodies against phos-mTOR and phos-ACC. B: BAEC were transduced with adenovirus vectors encoding SOD1 or catalase for 48 hrs and then treated with 5 mM 2-DG for 24 hrs. Cell lysates were analyzed by Western blot using antibodies against SOD1, catalase, and β-actin.

**
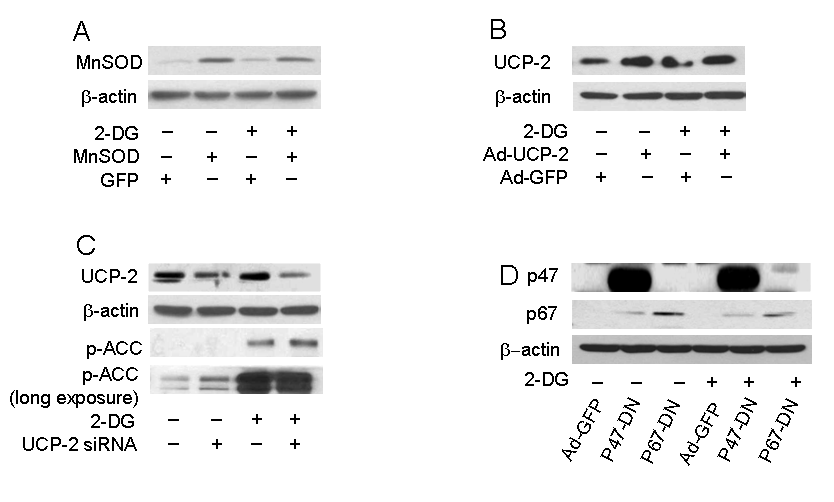
**

**Supplement Figure S3.**

**BAEC were transduced with adenovirus coding for MnSOD, UCP-2, p47 DN, p67-DN.** A, B, D: BAEC were transduced with adenovirus vectors encoding MnSOD (A), UCP2 (B), or p47-DN and p67-DN (D) for 48 hrs and then treated with 5 mM 2-DG for 10 min. Cell lysates were analyzed by Western blot analysis for MnSOD, UCP2, p47^phox^, p67^phox^, and β-actin. C: HUVEC were transfected UCP2-targeted siRNA or control siRNA for 24 hrs. Cell lysates were analyzed by Western blot analysis using antibodies against UCP2, p-ACC, and β-actin.
